# Dietary fibre and incidence of type 2 diabetes in eight European countries: the EPIC-InterAct Study and a meta-analysis of prospective studies

**DOI:** 10.1007/s00125-015-3585-9

**Published:** 2015-05-29

**Authors:** The InterAct Consortium

**Affiliations:** c/o A. Kuijsten, Division of Human Nutrition, Wageningen University, PO Box 8129, 6700 EV Wageningen, the Netherlands

**Keywords:** Case-cohort, Dietary fibre, EPIC-InterAct, Meta-analysis, Type 2 diabetes

## Abstract

**Aims/hypothesis:**

Intake of dietary fibre has been associated with a reduced risk of type 2 diabetes, but few European studies have been published on this. We evaluated the association between intake of dietary fibre and type 2 diabetes in the European Prospective Investigation into Cancer and Nutrition (EPIC)-InterAct study and in a meta-analysis of prospective studies.

**Methods:**

During 10.8 years of follow-up, 11,559 participants with type 2 diabetes were identified and a subcohort of 15,258 participants was selected for the case-cohort study. Country-specific HRs were estimated using Prentice-weighted Cox proportional hazards models and were pooled using a random effects meta-analysis. Eighteen other cohort studies were identified for the meta-analysis.

**Results:**

In the EPIC-InterAct Study, dietary fibre intake was associated with a lower risk of diabetes (HR_Q4 vs Q1_ 0.82; 95% CI 0.69, 0.97) after adjustment for lifestyle and dietary factors. Similar inverse associations were observed for the intake of cereal fibre and vegetable fibre, but not fruit fibre. The associations were attenuated and no longer statistically significant after adjustment for BMI. In the meta-analysis (19 cohorts), the summary RRs per 10 g/day increase in intake were 0.91 (95% CI 0.87, 0.96) for total fibre, 0.75 (95% CI 0.65, 0.86) for cereal fibre, 0.95 (95% CI 0.87, 1.03) for fruit fibre and 0.93 (95% CI 0.82, 1.05) for vegetable fibre.

**Conclusions/interpretation:**

The overall evidence indicates that the intake of total and cereal fibre is inversely related to the risk of type 2 diabetes. The results of the EPIC-InterAct Study suggest that the association may be partially explained by body weight.

**Electronic supplementary material:**

The online version of this article (doi:10.1007/s00125-015-3585-9) contains peer-reviewed but unedited supplementary material, which is available to authorised users.

## Introduction

Worldwide, there is an increasing prevalence of type 2 diabetes [1], which is likely to be driven by increasing adiposity, reduced physical activity and dietary changes. The number of people living with diabetes (mostly type 2 diabetes) worldwide has been projected to increase from 366 million in 2011 to 552 million by 2030 [1], and this trend will have important public health implications in terms of morbidity [2], mortality [2, 3] and healthcare costs [4].

The intake of dietary fibre, especially of cereal origin, has been inversely associated with risk of diabetes, as has been summarised in a meta-analysis of cohort studies by Schulze et al [5]. A higher intake of cereal fibre was associated with a 33% lower risk of diabetes compared with a low intake [5]. However, most of the studies included in this meta-analysis were from the USA, and the level and sources of fibre intake may differ substantially between countries. For example, in European populations [6, 7] total fibre intake appears to be higher than that reported in several US studies [8–10], and this may partly be explained by a higher intake of cereal fibre in Europe compared with the US [6, 8–10]. It is also not clear why cereal fibre could exert more beneficial effects on type 2 diabetes than other sources of fibre. Most cereals contain proportionally larger amounts of insoluble fibre, while most evidence from experimental studies on the benefits of fibre has been accumulated for soluble fibres [11]. The aims of this study were to evaluate the associations between total, cereal, fruit and vegetable fibre and the incidence of type 2 diabetes in a large European cohort, the European Prospective Investigation into Cancer and Nutrition (EPIC)-InterAct Study, and to summarise the existing evidence on fibre intake and type 2 diabetes in a meta-analysis of prospective studies.

## Methods

### Study population

The EPIC-InterAct Study is embedded in EPIC, which is a multicentre prospective cohort study designed to investigate the relationship between food habits, nutritional status, various lifestyle and environmental factors, and the incidence of cancer and other chronic diseases in ten European countries [12, 13]. The EPIC-InterAct Study used data from eight European countries (Denmark, France, Germany, Italy, the Netherlands, Spain, Sweden and the UK) [14]. We used a nested case-cohort design, including incident cases of type 2 diabetes (*n* = 12,403) and a random subcohort (*n* = 16,835, including 778 cases of incident diabetes), selected from 340,234 EPIC participants eligible for the EPIC-InterAct Study. All the participants gave written informed consent, and the study was approved by the local ethics committee at the participating centres and the Internal Review Board of the International Agency for Research on Cancer.

### Population for current analysis

Of the 28,460 participants in the EPIC-InterAct nested case-cohort sample, we excluded participants with prevalent diabetes (*n* = 548), missing information on reported diabetes status (*n* = 129), post-censoring diabetes (*n* = 4), extreme energy intake (in the top 1% and bottom 1% of the distribution of the ratio of reported energy intake over estimated energy requirement, assessed by basal metabolic rate; *n* = 736), and participants with missing values for educational level (*n* = 479), physical activity (*n* = 180), smoking status (*n* = 131) and BMI (*n* = 165). This left a final sample of 11,559 cases and 15,258 subcohort participants (729 of the diabetes cases being from the subcohort), for the current analysis (a total of 26,088 participants). No differences were observed in baseline characteristics between the included and excluded participants.

### Incidence of diabetes

Incident cases of diabetes were identified on the basis of self-reporting, a linkage to primary care registers or secondary care registers, medication use, hospital admissions and mortality data [14]. The identified cases were verified with further evidence, including individual medical record reviews. Cases in Denmark and Sweden were not ascertained by self-reporting but were identified via local and national diabetes and pharmaceutical registers. Follow-up was censored at the date of diagnosis, 31 December 2007 or the date of death, whichever occurred first. In total, 11,559 verified incident cases were identified during follow-up and were eligible for the current analysis.

### Fibre intake and other dietary variables

Dietary intake over the previous 12 months before recruitment was assessed by country-specific or centre-specific dietary assessment methods (food frequency questionnaires and dietary histories) that were developed and validated locally [12, 15, 16]. The food intake data were converted to nutrient intake using the European Nutrient Database [17]. The method for estimating total dietary fibre intake has previously been described [18]. In brief, the gravimetric method of the Association of Official Analytical Chemists [19] was used for estimating the total dietary fibre (which includes soluble and insoluble forms of non-starch polysaccharides and resistant starch as fibre) in all countries except the UK, where total dietary fibre was estimated by the Englyst method (which includes non-starch polysaccharides but not lignin or resistant starch). For the present study, we used measurements of total dietary fibre and fibre from cereals, fruits and vegetables, which are the main fibre sources in all eight countries.

### Lifestyle variables

Baseline information on lifestyle factors was obtained from questionnaires. Weight, height and waist circumference were measured by trained staff during standardised health checks at baseline in all centres, except for a proportion of participants in Oxford (UK) and France, for whom self-reported data were obtained, and Umeå (Sweden), where waist circumference was not measured. Physical activity was assessed by questionnaire and was classified according to the Cambridge Physical Activity Index [20, 21].

### Statistical analysis

We examined the association by country between quarters of the distribution of fibre intake in the subcohort (hereafter referred to as quartiles) adjusted for energy using the residual method [22] and the incidence of type 2 diabetes using Cox proportional hazard models modified for the case-cohort design according to the Prentice method [23]. The underlying time metric was age. In order to adjust for time to follow-up, the age at recruitment (1 year categories) was included as a stratum variable. Country-specific HRs and 95% CIs were pooled using random effects meta-analyses [24]. Between-country heterogeneity was assessed using the *I*^2^ statistic, i.e. the percentage of variation in the HR that was attributable to between-country heterogeneity [25]. The significance of linear trends across quartiles of total and different sources of fibre was tested by assigning the median value of the quartile to each participant and modelling these values as a continuous variable.

Confounders were assessed at baseline and those included in the models were age and sex (model 1), lifestyle and classical diabetes risk factors (model 2), dietary factors (model 3) and BMI (model 4). Each model was additionally adjusted for the preceding model. Lifestyle and classical diabetes risk factors included smoking status (never smoker, former smoker or current smoker), physical activity level based on an index of activity (inactive, moderately inactive, moderately active or active) [20, 21], education level (low, secondary or high) and alcohol intake (0 = non-drinker, 1 = 0–12/0–6 g/day for men and women, respectively, 2 = 12–24/6–12 g/day or 3 ≥ 24/12 g/day). Dietary factors included the total energy intake and the energy-adjusted intake of carbohydrates, magnesium, vitamin B_1_ and saturated fatty acids (continuous). The final model also adjusted for BMI (continuous). All models for subgroups of fibre were mutually adjusted for each other. Variables not included in the multivariable models because they did not change the risk estimates are listed in the electronic supplementary material (ESM) methods. A number of stratified and sensitivity analyses were conducted, and these are described in the ESM methods.

Analyses were conducted in SAS version 9.2 (SAS Institute, Cary, NC, USA), except for the meta-analyses, which were conducted in Stata 11.0 (Stata, College Station, TX, USA). A two-sided *p* value ≤0.05 was considered statistically significant for all analyses.

### Meta-analysis

We searched PubMed up to and including 24 January 2014 for prospective studies of fibre intake and risk of type 2 diabetes using the keywords ‘fiber’, ‘fibre’ and ‘diabetes’. Eighteen cohorts (20 publications) [5–10, 26–39] in addition to the present study were included in the analyses. More details of the study selection and methods can be found in the ESM methods. Random effects models were used to calculate summary RRs comparing the highest with the lowest category of fibre intake and for the dose–response analysis [24]. Dose–response analyses were conducted using the method described by Greenland and Longnecker [40]. Non-linear dose–response analyses were conducted using fractional polynomial models [41], and a likelihood ratio test was used to test for non-linearity [41]. We quantified the extent of heterogeneity by using *I*^2^ [25]. We tested for small-study bias using Egger’s test [42] and by inspecting the funnel plots. All statistical analyses for the meta-analysis were conducted using the statistical package STATA 11.0.

## Results

### The EPIC-InterAct Study

The study population consisted of 26,088 participants. The average age at baseline in the subcohort (*n* = 15,258) was 52.4 ± 9.1 years. Participants who had a high fibre intake (>26.4 g/day) were less likely to smoke, drank little alcohol and were more physically active than those with a low fibre intake (<18.9 g/day; Table 1). The proportion of men was higher in the lowest quartile of fibre intake (50% men) than in the next three quartiles (31–35%). Although the mean BMI was slightly higher in participants with a higher fibre intake (Table 1), this may have been confounded by country as fibre intake was positively associated with BMI only in Spain and the Netherlands, was not associated with BMI in Italy, and was inversely associated with BMI in the remaining countries (ESM Table 1).

**Table 1:** Study characteristics (mean ± SD, unless otherwise specified) in a random subcohort from the EPIC-InterAct study stratified by quartiles of energy-adjusted total fibre intake (*n* = 15,258)

Variable	*n*	Q1	Q2	Q3	Q4
Cutoffs (g/day)		<18.9	18.9–<22.4	22.4–26.4	>26.4
Median (g/day)		16.3	20.7	24.2	29.7
Age (years)	15,258	52.0 ± 9.4	52.2 ± 9.1	52.6 ± 9.0	52.8 ± 8.7
Men (%)	15,258	50	35	31	35
Follow-up (years)	15,258	12.0 ± 2.5	12.0 ± 2.3	11.9 ± 2.4	12.0 ± 2.2
BMI (kg/m^2^)	15,258	25.8 ± 4.0	26.0 ± 4.2	26.2 ± 4.2	26.2 ± 4.3
BMI (% obese)	15,258	14	16	17	16
Waist circumference (cm)					
Men	5,282	94.8 ± 10.2	94.9 ± 9.9	95.9 ± 9.6	95.1 ± 9.8
Women	8,963	80.1 ± 11.4	81.0 ± 10.9	81.7 ± 11.0	81.9 ± 11.3
First-degree relatives with diabetes (% yes)^a^	7,615	17	19	21	19
Smoking (% current)	15,258	37	26	23	18
Hypertension (% yes)	14,930	18	19	20	19
Hyperlipidaemia (% yes)	11,389	18	19	19	19
Myocardial infarction (% yes)	15,007	1.4	1.3	1.5	1.4
Angina (% yes)	10,078	1.7	1.8	2.3	2.4
Stroke (% yes)	14,036	0.9	0.9	0.8	0.8
Educational level (% high)	15,258	21	21	20	21
Physical activity (% inactive)	15,258	25	24	23	22
Postmenopausal women (%)	9,484	44	46	47	51
Dietary factors					
Energy (kJ/day)	15,258	9,320 ± 2,834	8,579 ± 2,562	8,629 ± 2,516	9,286 ± 2,617
Fat (en%)	15,258	36.7	35.4	34.3	32.7
Saturated fatty acids		14.8	13.6	12.8	11.7
Monounsaturated fatty acids		13.8	13.5	13.0	12.4
Polyunsaturated fatty acid		5.4	5.6	5.7	5.7
Protein (en%)	15,258	16.5	17.0	17.3	17.4
Carbohydrates (en%)	15,258	40.6	43.35	45.1	47.0
Starch		21.6	23.7	24.5	25.4
Sugars		18.4	19.1	20.0	20.9
Magnesium (mg/day)	15,258	313 ± 64	337 ± 59	362 ± 61	398 ± 65
Cholesterol (mg/day)	15,258	373 ± 125	352 ± 105	337 ± 104	314 ± 113
Vitamin B_1_ (mg/day)	15,258	1.2 ± 0.3	1.3 ± 0.3	1.4 ± 0.3	1.5 ± 0.4
β-Carotene (mg/day)	15,258	1.9 ± 1.3	2.6 ± 1.6	3.1 ± 1.9	4.4 ± 3.8
Vitamin C (mg/day)	15,258	88 ± 44	111 ± 45	131 ± 52	167 ± 83
Vitamin E (mg/day)	15,258	10.1 ± 4.1	11.5 ± 3.9	12.0 ± 4.1	13.1 ± 4.9
GI (not energy adjusted)	15,258	56.6 ± 4.1	55.9 ± 3.8	55.9 ± 3.8	55.8 ± 3.9
GL (not energy adjusted)	15,258	128 ± 47	124 ± 44	130 ± 43	145 ± 47
Alcohol (g/day) median (P10; P90), not energy adjusted	15,258	12 (0; 58)	7 (0; 37)	5 (0; 31)	4 (0; 30)
Alcohol (% non-drinkers)	15,258	19	26	29	31

^a^Family history of diabetes was not ascertained in Italy, Spain, Oxford and Heidelberg (excluded from this summary)

en%, percentage of total energy intake; GI, glycaemic index; GL, glycaemic load; P10, 10th percentile; P90, 90th percentile

The mean ± SD fibre intake in the subcohort was 22.9 ± 6.2 g/day (ranging from 19.9 g/day in Sweden to 25.2 g/day in Denmark; data not shown). Cereals were the main source of fibre (38%) in all countries except France, where vegetables were the main source of fibre. Of the cereal fibres, 81% originated from bread (ranging from 56% in the UK to 90% in Germany), 8% from pasta and rice (with the highest amounts in Italy [23%] and France [16%]) and 7% from breakfast cereals (with the highest amount in the UK [28%]). Other sources of fibre in consecutive order were fibre from fruits and nuts (20%), vegetables (18%), potatoes and tubers (9%) and legumes (5%). Cereal, fruit and vegetable fibres together accounted for around 75% of total fibre in all countries (data not shown).

**Fig. 1:**
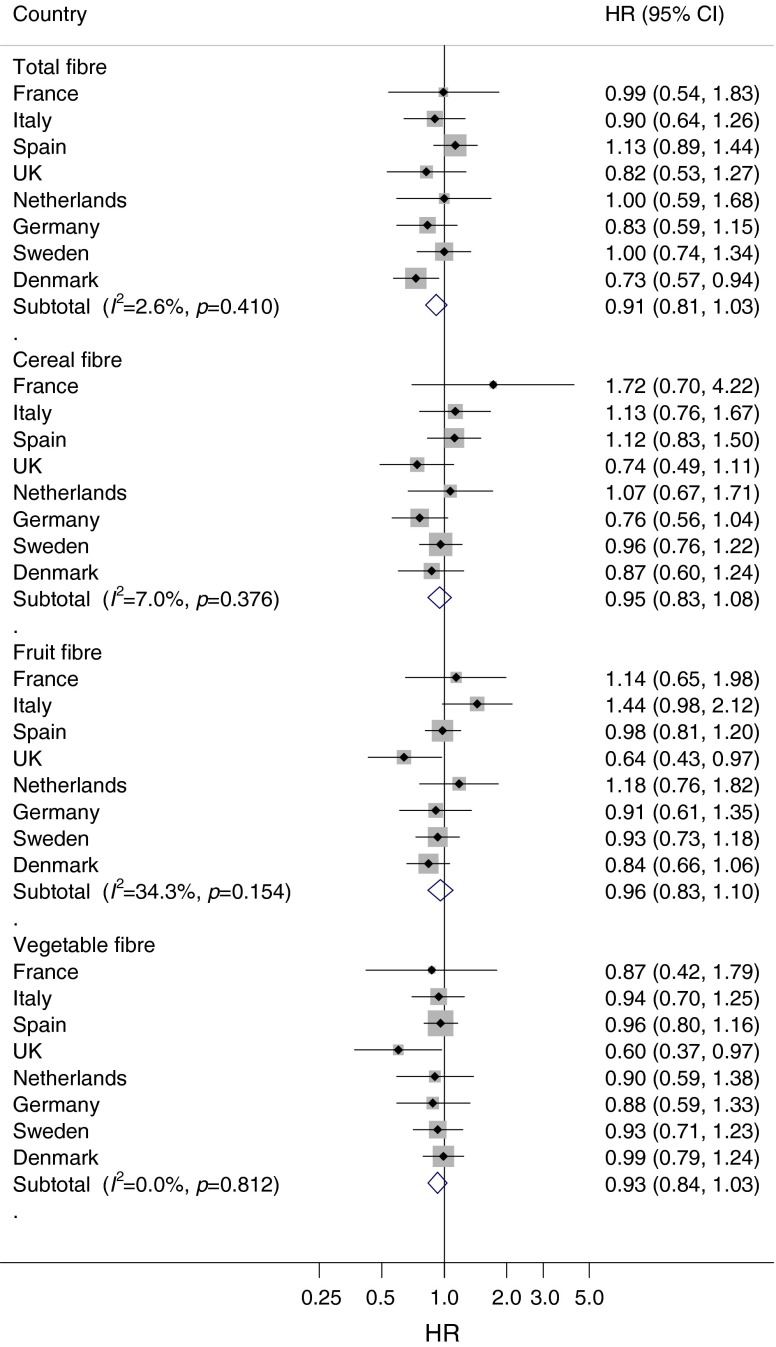
Association between cereal fibre, fruit fibre and vegetable fibre consumption and risk of type 2 diabetes in the EPIC-InterAct study (*n* = 26,088). Country-specific HR_Q4 vs Q1_ (95% CIs) were pooled using random effects meta-analysis. HRs were adjusted for sex, smoking status, physical activity, education level, sex-specific alcohol categories, energy, energy-adjusted carbohydrate, magnesium intake, vitamin B_1_ intake, saturated fatty acids and BMI. The *x*-axis is on a log scale

During a median of 10.8 years of follow-up, we ascertained 11,559 incident cases of type 2 diabetes. After adjusting for lifestyle factors and dietary factors, total fibre intake was associated with a lower risk of diabetes (HR_Q4 vs Q1_ 0.82; 95% CI 0.69, 0.97; *p* for trend = 0.02; Table 2). When evaluating the fibre sources, the highest vs the lowest quartile of intake of cereal fibre (HR 0.81; 95% CI 0.70, 0.93; *p* for trend <0.01) and vegetable fibre (HR 0.84; 95% CI 0.74, 0.96; *p* for trend <0.01) were inversely associated with the risk of diabetes, but fruit fibre (HR 0.98; 95% CI 0.89, 1.08; *p* for trend = 0.74) was not associated with risk of diabetes. However, when the analyses were additionally adjusted for BMI, the inverse associations were attenuated and no longer statistically significant. When comparing the highest with the lowest quartile, the HRs were 0.91 (95% CI 0.81, 1.03; *p* for trend = 0.28) for total fibre, 0.95 (95% CI 0.83, 1.08; *p* for trend = 0.49) for cereal fibre, 0.96 (95% CI 0.83, 1.10; *p* for trend = 0.76) for fruit fibre and 0.93 (95% CI 0.84, 1.03; *p* for trend = 0.11) for vegetable fibre. Adjustment for BMI explained 50% of the association between total fibre and type 2 diabetes. We did not observe substantial between-country heterogeneity for total fibre or sources of fibre (*I*^2^ = 2.6%, 7.0%, 34.3% and 0% for total, cereal, fruit and vegetable fibre, respectively; Fig. 1). The association between dietary fibre and type 2 diabetes was not modified by sex, BMI, physical activity, smoking, magnesium intake, vitamin B_1_ intake, glycaemic index or glycaemic load (all *p* > 0.05). The results were not materially altered in several sensitivity analyses (see ESM results).

**Table 2:** HRs (95% CI) for the associations between quartiles of dietary fibre and incident type 2 diabetes in the EPIC-InterAct Study (*n* = 26,088)

Variable	Q1	Q2	Q3	Q4	*p*
Total fibre, g/day (median)	<18.9 (16.3)	18.9–22.4 (20.7)	22.4–26.4 (24.2)	>26.4 (29.7)	
Model 1 ‘age, sex’	1.00	0.91 (0.82, 1.02)	0.90 (0.79, 1.02)	0.93 (0.78, 1.11)	0.08
Model 2 ‘lifestyle’	1.00	0.92 (0.82, 1.04)	0.91 (0.81, 1.02)	0.84 (0.69, 1.02)	0.07
Model 3 ‘lifestyle and diet’	1.00	0.92 (0.83, 1.02)	0.91 (0.82, 1.01)	0.82 (0.69, 0.97)	0.02
Model 4 ‘lifestyle, diet and BMI’	1.00	0.93 (0.84, 1.03)	0.98 (0.89, 1.08)	0.91 (0.81, 1.03)	0.28
Cereal fibre, g/day (median)	<5.7 (4.3)	5.7–7.9 (6.8)	7.9–10.9 (9.3)	>10.9 (13.7)	
Model 1 ‘age, sex’	1.00	0.89 (0.80, 0.99)	0.88 (0.77, 1.00)	0.85 (0.72, 1.00)	0.03
Model 2 ‘lifestyle’	1.00	0.89 (0.80, 0.98)	0.89 (0.80, 0.99)	0.82 (0.72, 0.93)	<0.01
Model 3 ‘lifestyle and diet’	1.00	0.90 (0.80, 1.00)	0.91 (0.82, 1.00)	0.81 (0.70, 0.93)	<0.01
Model 4 ‘lifestyle, diet, and BMI’	1.00	0.90 (0.80, 1.02)	1.00 (0.90, 1.10)	0.95 (0.83, 1.08)	0.49
Fruit fibre, g/day (median)	<2.3 (1.4)	2.3–4.0 (3.1)	4.0–6.6 (5.1)	>6.3 (8.4)	
Model 1 ‘age, sex’	1.00	0.88 (0.82, 0.95)	0.91 (0.83, 1.00)	0.89 (0.81, 0.98)	0.17
Model 2 ‘lifestyle’	1.00	0.91 (0.84, 0.98)	0.96 (0.88, 1.04)	0.93 (0.85, 1.01)	0.41
Model 3 ‘lifestyle and diet’	1.00	0.93 (0.86, 1.00)	0.98 (0.90, 1.06)	0.98 (0.89, 1.08)	0.74
Model 4 ‘lifestyle, diet, and BMI’	1.00	0.91 (0.82, 1.00)	0.93 (0.84, 1.03)	0.96 (0.83, 1.10)	0.76
Vegetable fibre, g/day (median)	<2.4 (1.6)	2.4–3.6 (3.0)	3.6–5.3 (4.4)	>5.3 (6.9)	
Model 1 ‘age, sex’	1.00	0.95 (0.88, 1.02)	0.87 (0.76, 0.98)	0.93 (0.78, 1.11)	0.57
Model 2 ‘lifestyle’	1.00	0.98 (0.91, 1.06)	0.91 (0.81, 1.03)	0.98 (0.85, 1.14)	0.92
Model 3 ‘lifestyle and diet’	1.00	0.94 (0.87, 1.01)	0.82 (0.71, 0.94)	0.84 (0.74, 0.96)	<0.01
Model 4 ‘lifestyle, diet, and BMI’	1.00	0.91 (0.84, 0.99)	0.83 (0.71, 0.97)	0.93 (0.84, 1.03)	0.11

Model 1 was adjusted for age and sex

Model 2 was additionally adjusted for lifestyle factors: smoking status, physical activity, education level and sex-specific alcohol categories

Model 3 was additionally adjusted for dietary factors: energy and energy-adjusted carbohydrates, magnesium, vitamin B_1_ and saturated fatty acids

Model 4 was additionally adjusted for BMI

All models for types of fibre were mutually adjusted

### Meta-analysis

Eighteen independent cohorts (20 publications) [5–10, 26–39] in addition to the EPIC-InterAct study were included in the meta-analysis, with a total of 617,968 participants and 41,066 incident cases of type 2 diabetes (Table 3, ESM Fig. 1). The study characteristics of the cohorts are provided in Table 3. Of the 19 cohort studies, eight were conducted in the USA, five in Europe, three in Australia and three in Asia.

**Table 3:** Prospective studies of dietary fibre intake and type 2 diabetes included in the meta-analysis

Author, publication year, country/ region	Study name	Population	Follow-up (years)	Age at baseline (range in years)	No. of cases	Dietary assessment	Exposure	Quantity	RR (95% CI)	Adjustment for confounders
Hodge et al, 1993, Australia [39]	NA	350 M/W	5	>20	7	24 h dietary recall	Total fibre	Per 10 g/day	0.69 (0.16, 2.96)	Age, sex, BMI, energy
Salmeron et al, 1997, USA [8]	Nurses’ Health Study	85,173 W	6	40–65	915	Validated FFQ, 134 food items	Total fibre	24.1 vs 11.8 g/day	0.78 (0.62, 0.98)	Age, BMI, alcohol, smoking, physical activity, family history of diabetes, energy
							Cereal fibre	7.5 vs 2.0 g/day	0.72 (0.58, 0.90)	
							Fruit fibre	7.6 vs 1.4 g/day	0.87 (0.70, 1.08)	
							Vegetable fibre	9.6 vs 3.4 g/day	1.17 (0.93, 1.46)	
Salmeron et al, 1997, USA [26]	Health Professionals Follow-up Study	42,759 M	6	40–75	523	Validated FFQ, 131 food items	Total fibre	29.7 vs 13.4 g/day	0.98 (0.73, 1.33)	Age, BMI, alcohol, smoking, physical activity, family history of diabetes, energy
							Cereal fibre	10.2 vs 2.5 g/day	0.70 (0.51, 0.96)	
							Fruit fibre	8.3 vs 1.2 g/day	1.01 (0.76, 1.36)	
							Vegetable fibre	11.3 vs 3.5 g/day	1.12 (0.84, 1.49)	
Meyer et al, 2000, USA [10]	Iowa Women’s Health	35,988 W	6	55–69	1,141	Validated FFQ, 127 food items	Total fibre	26.5 vs 13.27 g/day	0.78 (0.64, 0.96)	Age, BMI, WHR, alcohol, smoking, physical activity, education level, energy intake
							Cereal fibre	9.43 vs 2.66 g/day	0.64 (0.53, 0.79)	
							Fruit fibre	8.72 vs 1.71 g/day	1.17 (0.96, 1.42)	
							Vegetable fibre	11.74 vs 4.71 g/day	0.97 (0.80, 1.18)	
							Soluble fibre	8.01 vs 4.19 g/day	0.89 (0.73, 1.08)	
							Insoluble fibre	19.84 vs 9.93 g/day	0.75 (0.61, 0.91)	
Hu et al, 2001, USA [27]	Nurses’ Health Study	84,941 W	16	34–59	3,300	Validated FFQ, 61–120 food items	Cereal fibre	Quintile 5 vs 1	0.59 (0.52, 0.68)	Age, time, family history of diabetes, menopausal status, HT, smoking status, BMI, moderate-to-vigorous exercise, alcohol, TFA, PUFA/SFA ratio, GL
Stevens et al, 2002, USA [28]	Atherosclerosis Risk in Communities Study	9,529 M/W white participants, 2,722 M/W AA participants	9	45–64	971 and 478	Validated FFQ, 66 food items	Total fibre, whites	Per 1 g/day	0.999 (0.987, 1.012)	Age, sex, BMI, smoking, physical activity, education level, field centre, energy
							Cereal fibre, whites	Per 1 g/day	0.956 (0.925, 0.987)	
							Fruit fibre, whites	Per 1 g/day	1.002 (0.983, 1.021)	
							Total fibre, AA	Per 1 g/day	0.998 (0.980, 1.017)	
							Cereal fibre, AA	Per 1 g/day	0.982 (0.927, 1.039)	
							Fruit fibre, AA	Per 1 g/day	1.009 (0.985, 1.033)	
							Cereal fibre, whites	Quintile 5 vs 1	0.75 (0.60, 0.92)	
							Cereal fibre, AA	Quintile 5 vs 1	0.86 (0.65, 1.15)	
Montonen et al, 2003, Finland [6]	Finnish Mobile Clinic Health Examination Survey	4,318 M/W	10	40–69	156	FFQ, 100 food items	Total fibre	≥33.2 vs ≤19.2 g/day	0.51 (0.26, 1.00)	Age, sex, geographical area, smoking BMI, intakes of energy, fruit and berries, vegetables
							Cereal fibre	≥24.5 vs ≤12.0 g/day	0.39 (0.20, 0.77)	
							Fruit fibre	≥3.4 vs ≤0.99 g/day	0.92 (0.40, 2.13)	
							Vegetable fibre	≥26.5 vs ≤3.7 g/day	1.19 (0.46, 3.04)	
							Soluble fibre	≥7.4 vs ≤4.5 g/day	0.57 (0.29, 1.12)	
							Insoluble fibre	≥16.6 vs ≤8.7 g/day	0.47 (0.25, 0.91)	
Schulze et al, 2004, USA [9]	Nurses’ Health Study II	91,249 W	8	26–46	741	Validated FFQ, 133 food items	Total fibre	24.9 vs 12.5 g/day	1.00 (0.75, 1.34)	Age, BMI, alcohol, smoking, physical activity, family history of diabetes, high blood pressure, high blood cholesterol, HT, OC use, energy intake, Mg, and caffeine, GL, mutual adjustment between fibre types
							Cereal fibre	8.8 vs 3.1 g/day	0.64 (0.48, 0.86)	
							Fruit fibre	6.2 vs 1.1 g/day	0.79 (0.60, 1.02)	
							Vegetable fibre	10.4 vs 3.4 g/day	1.12 (0.87, 1.46)	
Hodge et al, 2004, Australia [29]	Melbourne Collaborative Cohort Study	31,641 M/W	4	40–69	365	FFQ, 121 food items	Fibre	Per 20 g/day	1.02 (0.81, 1.30)	Age, sex, BMI, WHR, weight change, alcohol, smoking, physical activity, family history of diabetes, education, country of birth, energy intake
							Cereal fibre	Per 10 g/day	1.08 (0.88, 1.32)	
							Fruit fibre	Per 10 g/day	0.97 (0.81, 1.16)	
							Vegetable fibre	Per 5 g/day	1.00 (0.86, 1.17)	
Lindström et al, 2006, Finland [7]	The Finnish Diabetes Prevention Study	172 M, 350 W	4.1	40–64	114	3 day food record	Fibre	>15.55 vs <10.85 g/4.1868 MJ/day	0.38 (0.19, 0.77)	Age, sex, baseline weight, baseline 2 h glucose, physical activity, weight change, energy
Barclay et al, 2007, Australia [30]	Blue Mountains Eye Study	1,833 M/W	10	≥49	138	Validated FFQ, 145 food items	Fibre	Per 5 g/day	0.90 (0.79, 1.02)	Age, sex, smoking, physical activity, family history of diabetes, triacylglycerols, HDL cholesterol
							Cereal fibre	Per 5 g/day	0.96 (0.78, 1.20)	
							Fruit fibre	Per 5 g/day	0.94 (0.78, 1.15)	
							Vegetable fibre	Per 5 g/day	0.76 (0.57, 0.99)	
Krishnan et al, 2007, USA [31]	Black Women’s Health Study	40,078 W	8	21–69	1,938	Validated FFQ, 68 food items	Cereal fibre	7.6 vs 1.7 g/day	0.82 (0.70, 0.96)	Age, BMI, smoking, physical activity, family history of diabetes, total fat, protein intake, GI, energy intake
Schulze et al, 2007, Germany [5]	EPIC-Potsdam	9,702 M, 15,365 W	7	35–65	844	Validated FFQ	Soluble fibre	9.6 vs 5.3 g/day	0.83 (0.57, 1.22)	Age, sex, BMI, sports activities, education, cycling, occupational activity, smoking, alcohol, total energy intake, waist circumference, PUFA:SFA ratio, MUFA:SFA ratio, carbohydrate, Mg
							Insoluble fibre	18.4 vs 10.3 g/day	0.93 (0.62, 1.40)	
Wannamethee et al, 2009, UK [32]	British Regional Heart Study	3,428 M	7	60–79	162	Validated 7 day recall FFQ	Total fibre	≥31.0 vs ≤20 g/day	0.82 (0.51, 1.32)	Age, waist circumference, alcohol, smoking, physical activity, social class, pre-existing MI or stroke, statin use, energy intake
							Cereal fibre	Quartile 4 vs 1	0.70 (0.44, 1.12)	
							Vegetable fibre	Quartile 4 vs 1	0.74 (0.46, 1.19)	
Hopping et al, 2010, USA [33]	Multiethnic cohort	36,256 M, 39,256 W	14	45–75	4,555 and 4,032	Validated FFQ	Total fibre, M	14.2 vs 7.4 g/4.1868 MJ/day	0.75 (0.67, 0.84)	Age, BMI, physical activity, education, ethnicity, energy intake
							Cereal fibre, M	4.8 vs <1.9 g/4.1868 MJ/day	0.91 (0.82, 1.00)	
							Fruit fibre, M	3.9 vs <0.8 g/4.1868 MJ/day	0.93 (0.84, 1.02)	
							Vegetable fibre, M	5.3 vs <2.2 g/4.1868 MJ/day	0.78 (0.69, 0.88)	
							Total fibre, W	16.2 vs <8.9 g/4.1868 MJ/day	0.95 (0.85, 1.06)	
							Cereal fibre, W	5.1 vs <2.1 g/4.1868 MJ/day	0.88 (0.79, 0.97)	
							Fruit fibre, W	5.1 vs <2.1 g/4.1868 MJ/day	0.95 (0.85, 1.06)	
							Vegetable fibre, W	5.2 vs <1.3 g/4.1868 MJ/day	0.96 (0.87, 1.08)	
Sakurai et al, 2012, Japan [34]	NA	1,995 M	6	35–55	133	DHQ, 147 items	Fibre	>6.0 vs <3.7 g/day	0.99 (0.59, 1.66)	Age, BMI, family history of diabetes, exercise, hypertension, hyperlipidaemia, total energy, GI, GL
Wirström et al, 2013, Sweden [35]	NA	5,477 M/W	8–10	35–56	165	Validated FFQ, NA	Cereal fibre	Per 10 g/day	0.97 (0.82, 1.14)	Age, family history of diabetes, BMI, leisure-time physical activity, smoking, education, blood pressure
								>11.6 vs <7.7 g/day	1.02 (0.68, 1.52)	
Liu et al, 2012, China [37]	NA	3,461 M/W	>5	35–74	162	NA	Total fibre	High vs low	0.38 (0.17, 0.87)	Age, sex, family history of diabetes, blood pressure, triacylglycerols, fasting glucose
Weng et al, 2012, Taiwan [36]	CardioVascular Disease risk FACtor Two-township Study	1,604 M/W	4.6	>30	141	Validated FFQ, 49 food items	Total fibre	43.3 vs 20.4 g/day	0.49 (0.28, 0.85)^a^	Age, sex, age–sex interaction, calories, residential area, family history of diabetes, BMI, central obesity, smoking, drinking, physical activity, hypertension, hypercholesterolaemia, hypertriacylglycerolaemia, low HDL-cholesterol
							Fruit fibre	20.6 vs 3.1 g/day	0.55 (0.32, 0.95)^a^	
							Vegetable fibre	15.8 vs 3.2 g/day	0.45 (0.25, 0.82)^a^	
Qiao et al, 2014, USA [38]	Women’s Health Initiative	154,493 W	7.6	50–79	10,285	Validated FFQ	Total fibre	≥13.14 vs <13.14 g/day	0.98 (0.93, 1.04)	Age, education, cigarette smoking, BMI, WHR, physical activity, family history of diabetes, study arms, HT use
Present study, Europe	EPIC-InterAct	26,088 M/W	10.8	20–79	11,559	Validated FFQs, diet history, 7 day food diaries	Total fibre	>26.4 vs <18.9 g/day	0.91 (0.81, 1.03)	Age, sex, smoking status, physical activity, education level, alcohol, energy intake, carbohydrates, Mg, vitamin B1, SFA, BMI
							Cereal fibre	>10.9 vs <5.7 g/day	0.95 (0.83, 1.08)	
							Fruit fibre	>6.3 vs <2.3 g/day	0.96 (0.83, 1.10)	
							Vegetable fibre	>5.3 vs <2.4 g/day	0.93 (0.84, 1.03)	

4.1868 MJ/day = 1,000 kcal/day

^a^Risk estimates provided in the article were for a low vs high (reference) comparison. For consistency with the remaining studies, these risk estimates have been converted so the comparison is for the highest vs the lowest intake

AA, African-American, DHQ, diet history questionnaire; FFQ, food frequency questionnaire; GI, glycaemic index; GL, glycaemic load; HT, hormone therapy; M, men; MI, myocardial infarction; MUFA, monounsaturated fatty acids; OC, oral contraceptive; PUFA, polyunsaturated fatty acids; SFA, saturated fatty acids; TFA, *trans* fatty acids; W; women

#### Total fibre

**Fig. 2:**
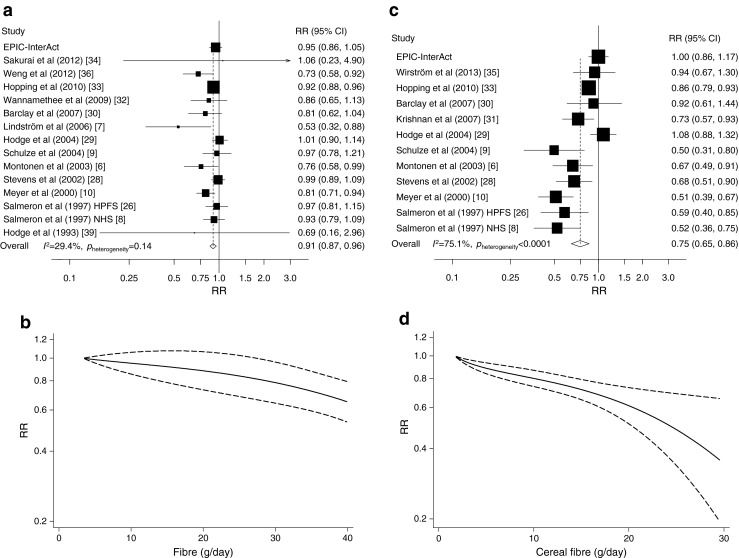
Dietary total fibre (**a**, **b**) and cereal fibre (**c**, **d**) and type 2 diabetes, linear dose–response meta-analyses per 10 g/day (**a**, **c**) and non-linear dose–response meta-analyses (**b**, **d**). In (**a**) and (**c**), the RR of each study is represented by a square, and the size of the square represents the weight of each study to the overall estimate. The 95% CIs are represented by horizontal lines, and the diamond represents the overall estimate and its 95% CI. The *x*-axis is on a log scale. In (**b**) and (**d**), the solid lines represent the best-fitting fractional polynomial, and the dashed lines represent 95% CIs

Sixteen studies [6–10, 26, 28–30, 32–34, 36–39] in addition to the EPIC-InterAct Study were included in the analysis of total fibre and type 2 diabetes (36,578 cases among 572,665 participants). Two of the studies [37, 38] were only included in the analysis of the highest vs the lowest intake. The summary RR comparing the highest vs the lowest intake was 0.85 (95% CI 0.77, 0.94; *I*^2^ 61.0%, *p*_heterogeneity_ = 0.002, *n* = 13) (ESM Fig. 2a), and in the dose–response analysis the summary RR per 10 g/day was 0.91 (95% CI 0.87, 0.96; *I*^2^ 29.4%, *p*_heterogeneity_ = 0.14, *n* = 15) (Fig. 2a). There was no evidence of non-linearity, with *p*_non-linearity_ = 0.37 (Fig. 2b, ESM Table 2).

#### Cereal fibre

Twelve studies (13 publications) [6, 8–10, 26–33, 35] in addition to the EPIC-InterAct Study were included in the analysis (30,224 cases and 455,563 participants). One of the studies [32] was only included in the analysis of the highest vs the lowest intake. The summary RR for the highest vs the lowest cereal fibre intake was 0.77 (95% CI 0.68, 0.87; *I*^2^ 77.7%, *p*_heterogeneity_ < 0.0001, *n* = 12) (ESM Fig. 2b) and per 10 g/day was 0.75 (95% CI 0.65, 0.86; *I*^2^ = 75.1%, *p*_heterogeneity_ < 0.0001, *n* = 12) (Fig. 2c). There was evidence of non-linearity (*p*_non-linearity_ = 0.004), with a steeper reduction in risk at higher levels of fibre intake (Fig. 2d, ESM Table 2).

#### Fruit fibre

**Fig. 3:**
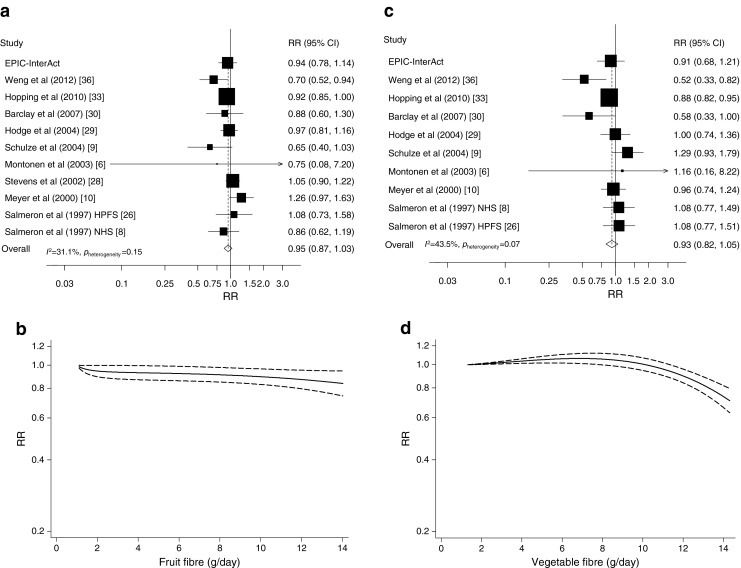
Fruit fibre (**a**, **b**) and vegetable fibre (**c**, **d**) and type 2 diabetes, linear dose–response meta-analyses per 10 g/day (**a**, **c**) and non-linear dose–response meta-analyses (**b**, **d**). In (**a**) and (**c**), the RR of each study is represented by a square and the size of the square represents the weight of each study to the overall estimate. The 95% CIs are represented by horizontal lines, and the diamond represents the overall estimate and its 95% CI. The *x*-axis is on a log scale. In (**b**) and (**d**), the solid lines represent the best-fitting fractional polynomial, and the dashed lines represent 95% CIs

Ten studies [6, 8–10, 26, 28–30, 33, 36] in addition to the EPIC-InterAct Study were included in the analysis (25,715 cases among 408,416 participants). The summary RR for the highest vs the lowest intake of fruit fibre was 0.95 (95% CI 0.88, 1.01; *I*^2^ 16.9%, *p*_heterogeneity_ = 0.29, *n* = 10) (ESM Fig. 2c) and per 10 g/day was 0.95 (95% CI 0.87, 1.03, *I*^2^ 31.1%, *p*_heterogeneity_ = 0.15, *n* = 11) (Fig. 3a). There was a suggestive non-linear association between fruit fibre and risk of type 2 diabetes (*p*_non-linearity_ = 0.04), with a slightly steeper curve when increasing intake from low levels, but the association was very weak (Fig. 3b, ESM Table 2).

#### Vegetable fibre

Ten studies [6, 8–10, 26, 29, 30, 32, 33, 36] in addition to the EPIC-InterAct Study were included in the analysis (24,428 cases among 399,593 participants). One of the studies [32] was only included in the analysis of the highest vs the lowest intake. The summary RR for the highest vs the lowest intake was 0.96 (95% CI 0.86, 1.07; *I*^2^ 48.3%, *p*_heterogeneity_ = 0.04, *n* = 10) (ESM Fig. 2d) and per 10 g/day was 0.93 (95% CI 0.82, 1.05; *I*^2^ = 43.5%, *p*_heterogeneity_ = 0.07, *n* = 10) (Fig. 3c). There was evidence of a non-linear association between vegetable fibre and risk of type 2 diabetes, (*p*_non-linearity_ < 0.0001), with an inverse association restricted to a very high intake (12–14 g/day) (Fig. 3d, ESM Table 2).

#### Soluble and insoluble fibre

Only three studies [5, 6, 10] investigated the intake of soluble and insoluble fibre and risk of diabetes (2,141 cases among 65,373 participants). The summary RR for the highest vs the lowest intake was 0.85 (95% CI 0.72, 1.01; *I*^2^ = 41.9%, *p*_heterogeneity_ = 0.18) for soluble fibre and 0.75 (95% CI 0.57, 0.97; *I*^2^ = 0%, *p*_heterogeneity_ = 0.44) (ESM Fig. 3a, b) for insoluble fibre. In the dose–response analysis, the summary RR per 10 g/day was 0.70 (95% CI 0.47, 1.04; *I*^2^ = 0%, *p*_heterogeneity_ = 0.50) for soluble fibre and 0.73 (95% CI 0.62, 0.86; *I*^2^ = 0%, *p*_heterogeneity_ = 0.46) for insoluble fibre (ESM Fig. 3c, d).

#### Subgroup, sensitivity analyses and publication bias

The results were in general consistent across the strata in the subgroup (ESM Tables 3 and 4) and sensitivity analyses (ESM results). Most of the studies adjusted for BMI, and the results persisted among studies that adjusted for BMI (ESM Tables 3 and 4). In the analysis of cereal fibre there was a suggestion of small-study bias with Egger’s test (*p* = 0.08) and asymmetry in the funnel plot suggesting that smaller studies with positive associations were missing (ESM Fig. 4). When restricting the analysis to four studies [10, 28, 31, 33] and the EPIC-InterAct study that had ≥1,000 cases, Egger’s test was no longer significant (*p* = 0.25), but the inverse association was similar to the overall analysis (summary RR 0.76; 95% CI 0.63, 0.92; *I*^2^ = 81.0%, *p*_heterogeneity_ < 0.0001). There was no evidence of publication bias for total fibre, fruit fibre or vegetable fibre (*p* = 0.16, *p* = 0.73 and *p* = 0.74, respectively).

## Discussion

The EPIC-InterAct study showed that a high intake of total fibre compared with a low intake was associated with an 18% lower risk of incident type 2 diabetes when adjusted for lifestyle and dietary factors. This was mainly driven by the intake of cereal fibre and vegetable fibre, and not by fruit fibre. When the results were adjusted for BMI, total fibre and cereal and vegetable fibre were not significantly associated with risk of type 2 diabetes. However, the findings from our updated meta-analysis of prospective studies do support an inverse association between total fibre and cereal fibre intake and risk of type 2 diabetes, with a 9% and 25% lower RR per 10 g/day, respectively, independent of BMI. A stronger inverse association between cereal fibre intake and type 2 diabetes than for fruit or vegetable fibre is consistent with previous meta-analyses of fibre intake and type 2 diabetes [5, 43], and with recent meta-analyses that have shown stronger associations for whole grain intake [44] than for fruit and vegetable intake in relation to risk of type 2 diabetes [45]. Differences in the strength and shape of the dose–response relationship compared with the previous meta-analyses [5, 43] may be due to the larger number of studies that was included in the present dose–response analyses and the addition of the EPIC-InterAct data. For example, in the linear dose–response analysis of dietary fibre, we included seven additional studies [6–8, 26, 33, 34, 39] as well as the present EPIC-InterAct study.

It has been suggested that the beneficial effect of cereal fibre observed in many studies could be explained by other nutrients co-ingested with the fibre, for example magnesium and vitamins such as B_1_, C and E [46]. In the EPIC-InterAct study, adding these nutrients to the models did not materially alter the association with cereal fibre. It is also possible that the low glycaemic index of diets high in total or cereal fibre could explain the relationship between fibre intake and diabetes. A low glycaemic index could lead to a lower postprandial glucose peak, which leads to a decreased insulin demand and protects the pancreas from exhaustion [47]. However, no association was observed between the glycaemic index or glycaemic load and diabetes in the EPIC-InterAct study [48], and further adjustment for both glycaemic index and glycaemic load did not change our results. This is consistent with other studies that have found little impact of additional adjustments for glycaemic index, glycaemic load and/or magnesium intake [9, 31, 34]. The intake of fruit fibre was not associated with type 2 diabetes in any of the models, but it is not clear why this is as the range of fruit fibre intake was comparable to that of other studies.

Our study has some limitations that could have affected the results. Measurement error in the assessment of dietary intake by questionnaire may have attenuated an association between fibre intake and type 2 diabetes. Different degrees of measurement error in the assessment of subtypes of fibre intake might explain the different magnitude of association observed with these subtypes. Dietary intake was assessed only at baseline, so we were not able to take into account dietary changes during follow-up. The strengths of the EPIC-InterAct study include the prospective design, the large number of cases, the extensive and validated dietary questionnaires, the wide range of dietary fibre intake in eight countries with a large variation in the different sources of fibre intake, and the detailed information on other potential confounders, including height and weight, which were measured in most of the study participants and may have reduced potential confounding by adiposity.

We cannot exclude the possibility that the inverse associations for total fibre and cereal fibre intake in the meta-analysis could be due to residual confounding as fibre intake has been associated with a healthier overall dietary pattern, a lower BMI and higher physical activity [6, 9, 10, 31]. Although most studies adjusted for BMI, physical activity, alcohol, smoking and energy intake, relatively few studies adjusted for other dietary factors. However, in the EPIC-InterAct study, adjustment for other dietary factors did not substantially alter the risk estimates. It is not clear why our result differs from the result of the meta-analysis. Weight and height were measured (rather than self-reported) in EPIC-InterAct. In general, adjustment for confounding with an imperfect measure of that confounder leads to the possibility of residual confounding. This is possible in this context as some other studies have used more imprecise measures, such as self-reported BMI, and could therefore have more issues with residual confounding. Of 11 studies of cereal fibre that adjusted for BMI, all six studies with self-reported weight and height reported inverse associations [8–10, 26, 31, 33], while only two [6, 28] out of five of the studies [6, 28, 29, 35] (including EPIC-InterAct) with measured weight and height reported significant inverse associations. However, data for other fibre types and total fibre do not appear to vary by whether weight and height was measured or self-reported, so chance can also not be excluded as an explanation.

In the meta-analysis of cereal fibre, there was some suggestion of small-study bias. However, when the analysis was restricted to studies with a large number of cases (≥1,000), there was no evidence of asymmetry in the funnel plot and Egger’s test was no longer significant, although the summary estimate was similar to that of the overall analysis.

The attenuation of the inverse associations we observed between total fibre, cereal fibre and vegetable fibre and diabetes after adjustment for BMI in the EPIC-InterAct analysis suggests that the beneficial effect of fibre may be partly mediated by a lower BMI, and this is consistent with other studies [5, 33]. A previous analysis in the EPIC study found an inverse association between fibre intake, particularly cereal fibre intake, and changes in weight and waist circumference [49], and other studies have also shown an inverse association between fibre intake and overweight, obesity, weight gain or visceral adiposity [50–54], although the data are not convincing [55]. However, as the observed effects of fibre intake on adiposity and weight change are relatively modest, BMI may act as both a confounder and a mediator in the relationship between fibre intake and diabetes. Dietary fibre may affect appetite and energy intake through a range of processes including a delayed emptying rate, a prolonged release of hormonal signals, a slowing of nutrient absorption or altered fermentation in the large intestine [51, 56].

Apart from reduced adiposity, dietary fibre may affect the risk of diabetes by other mechanisms as well. Dietary fibre intake improves glycaemic control by decreasing postprandial glycaemia and insulinaemia, and increases insulin sensitivity [57, 58]. There is also a cross-sectional association between the consumption of high-fibre breakfasts and markers of diabetes risk in children [59]. The fermentation of dietary fibres in the large intestine may alter the growth of specific gut bacteria, affect the production and composition of short-chain fatty acids and thereby affect the secretion of appetite-regulating peptides [60]. Furthermore, fermentable fibres may regulate the uptake of energy from the gut by the production or activation of signalling molecules involved in the host’s metabolism, a modification of gut permeability, the release of gut hormones and inflammation [61]. Based on intervention studies, the effect on glycaemic control appears to be stronger for soluble fibre than for insoluble fibre [62, 63], while in the meta-analysis we found an association with insoluble fibre and cereal fibre (which is high in insoluble fibre), but not with soluble fibre. Limited statistical power because of the low number of studies might explain the lack of association for soluble fibre as the risk estimates were of similar size, but further studies are needed to clarify whether there is a difference in the association between soluble and insoluble fibre and risk of diabetes.

In several, but not all, studies [64, 65], dietary fibre, cereal fibre and whole grains have been associated with lower concentrations of inflammatory markers [66–69], serum uric acid [70] and γ-glutamyltransferase [67, 71], markers that have been associated with increased risk of diabetes [72–74] and higher concentrations of adiponectin [75–77], an adipocyte-secreted cytokine that increases insulin sensitivity and may reduce risk of diabetes [78]. Alternatively, it is possible that other components of foods rich in cereal fibre such as whole grains could contribute to the reduced risk of diabetes by as yet unidentified mechanisms.

In summary, inverse associations were observed between the intake of total, cereal and vegetable fibre and risk of type 2 diabetes in the EPIC-InterAct study, but these associations were no longer significant after adjustment for BMI. In an up-to-date meta-analysis of all published prospective studies, we found an inverse association between total fibre and cereal fibre and risk of type 2 diabetes independent of BMI. Taken together, the results indicate that individuals with a diet rich in fibre, especially cereal fibre, may have a lower risk of type 2 diabetes.

### Electronic supplementary material

ESM Methods (PDF 85 kb)

ESM Results (PDF 53.9 kb)

ESM Figure 1 (PDF 51 kb)

ESM Figure 2 (PDF 145 kb)

ESM Figure 3 (PDF 170 kb)

ESM Figure 4 (PDF 8 kb)

ESM Table 1 (PDF 10 kb)

ESM Table 2 (PDF 15 kb)

ESM Table 3 (PDF 122 kb)

ESM Table 4 (PDF 102 kb)

## Appendix

The InterAct Consortium list of authors is as follows: Anneleen Kuijsten^†^ (Division of Human Nutrition, Wageningen University, Wageningen, the Netherlands); Dagfinn Aune^†^ (Department of Public Health and General Practice, Faculty of Medicine, Norwegian University of Science and Technology, Trondheim, Norway, and Department of Epidemiology and Biostatistics, School of Public Health, Imperial College, London, UK); Matthias B. Schulze (Department of Molecular Epidemiology, German Institute of Human Nutrition Potsdam-Rehbruecke, Nuthetal, Germany); Teresa Norat (Department of Epidemiology and Biostatistics, School of Public Health, Imperial College, London, UK); Geertruida J. van Woudenbergh (Division of Human Nutrition, Wageningen University, Wageningen, the Netherlands); Joline W. J. Beulens (Julius Center for Health Sciences and Primary Care, University Medical Center Utrecht, Utrecht, the Netherlands); Ivonne Sluijs (Julius Center for Health Sciences and Primary Care, University Medical Center Utrecht, Utrecht, the Netherlands); Annemieke M. W. Spijkerman (National Institute for Public Health and the Environment [RIVM], Bilthoven, the Netherlands); Daphne L. van der A (National Institute for Public Health and the Environment [RIVM], Bilthoven, the Netherlands); Domenico Palli (Molecular and Nutritional Epidemiology Unit, Cancer Research and Prevention Institute – ISPO, Florence, Italy); Tilman Kühn (Division of Cancer Epidemiology, German Cancer Research Center [DKFZ], Heidelberg, Germany); Andrea Wendt (Division of Cancer Epidemiology, German Cancer Research Center [DKFZ], Heidelberg, Germany); Brian Buijsse (Department of Epidemiology, German Institute of Human Nutrition Potsdam-Rehbruecke, Nuthetal, Germany); Heiner Boeing (Department of Epidemiology, German Institute of Human Nutrition Potsdam-Rehbruecke, Nuthetal, Germany); Valeria Pala (Epidemiology and Prevention Unit, Fondazione IRCCS Istituto Nazionale dei Tumori, Milano, Italy); Pilar Amiano (Public Health Division of Gipuzkoa, BioDonostia Research Institute, Health Department of Basque Region, San Sebastian, Spain, and CIBER Epidemiologia y Salud Publica [CIBERESP], Spain, www.ciberesp.es/); Genevieve Buckland (Unit of Nutrition, Environment and Cancer, Cancer Epidemiology Research Programme, Catalan Institute of Oncology [ICO-IDIBELL], Barcelona, Spain); José María Huerta Castaño (CIBERESP, Spain, www.ciberesp.es, and Department of Epidemiology, Murcia Regional Health Council, Murcia, Spain); Anne Tjønneland (Danish Cancer Society Research Center, Copenhagen, Denmark); Cecilie Kyrø (Danish Cancer Society Research Center, Copenhagen, Denmark); Maria Luisa Redondo (Public Health Directorate, Asturias, Spain); Carlotta Sacerdote (Center for Cancer Prevention (CPO-Piemonte), and Human Genetic Foundation [HuGeF], Torino, Italy); María-José Sánchez (Unit of Nutrition, Environment and Cancer, Cancer Epidemiology Research Programme, Catalan Institute of Oncology [ICO-IDIBELL], Barcelona, Spain, and Escuela Andaluza de Salud Pública, Instituto de Investigación Biosanitaria de Granada [Granada.ibs], Granada, Spain); Guy Fagherazzi (Inserm, Center for Research in Epidemiology and Population Health [CESP], U1018, Villejuif, France, and Paris-South University, UMRS 1018, Villejuif, France); Beverley Balkau (Inserm, Center for Research in Epidemiology and Population Health [CESP], U1018, Villejuif, France, and Paris-South University, UMRS 1018, Villejuif, France); Martin Lajous (Inserm, Center for Research in Epidemiology and Population Health [CESP], U1018, Villejuif, France, and Department of Epidemiology, Harvard School of Public Health, Boston, MA, USA, and Center for Research on Population Health, National Institute of Public Health, Cuernavaca, Mexico); Salvatore Panico (Dipartimento de Medicina Clinica e Chirurgia, Federico II University, Naples, Italy); Paul W. Franks (Department of Public Health and Clinical Medicine, Umeå University, Umeå, Sweden, and Department of Clinical Sciences, Lund University, Malmö, Sweden); Olov Rolandsson (Department of Public Health and Clinical Medicine, Umeå University, Umeå, Sweden); Peter Nilsson (Department of Clinical Sciences, Lund University, Malmö, Sweden); Marju Orho-Melander (Department of Clinical Sciences, Lund University, Malmö, Sweden); Kim Overvad (Department of Public Health, Section for Epidemiology, Aarhus University, Denmark); Inge Huybrechts (Dietary Exposure Assessment Group, International Agency for Research on Cancer, Lyon, France); Nadia Slimani (Dietary Exposure Assessment Group, International Agency for Research on Cancer, Lyon, France); Rosario Tumino (Cancer Registry and Histopathology Unit, ‘Civic – M.P.Arezzo’ Hospital, Ragusa, Italy); Aurelio Barricarte (CIBERESP, Spain, www.ciberesp.es/, and Navarre Public Health Institute, Pamplona, Spain); Timothy J. Key (Cancer Epidemiology Unit, Nuffield Department of Population Health, University of Oxford, Oxford, UK); Edith J. M. Feskens (Division of Human Nutrition, Wageningen University, Wageningen, the Netherlands); Claudia Langenberg (MRC Epidemiology Unit, University of Cambridge, School of Clinical Medicine, Institute of Metabolic Science, Cambridge Biomedical Campus, Cambridge, UK); Stephen Sharp (MRC Epidemiology Unit, University of Cambridge, School of Clinical Medicine, Institute of Metabolic Science, Cambridge Biomedical Campus, Cambridge, UK); Nita G. Forouhi (MRC Epidemiology Unit, University of Cambridge, School of Clinical Medicine, Institute of Metabolic Science, Cambridge Biomedical Campus, Cambridge, UK); Elio Riboli (Department of Epidemiology and Biostatistics, School of Public Health, Imperial College, London, UK); Nicholas J. Wareham (MRC Epidemiology Unit, University of Cambridge, School of Clinical Medicine, Institute of Metabolic Science, Cambridge Biomedical Campus, Cambridge, UK).

^†^ Joint first authors.

## Abbreviation

EPIC: European Prospective Investigation into Nutrition and Cancer
