# Supplementary material for: Dietary fibre and incidence of type 2 diabetes in eight European countries: the EPIC-InterAct Study and a meta-analysis of prospective studies
Source: Diabetologia. 2015 May 29;58(7):1394–408. doi: 10.1007/s00125-015-3585-9 (PMC4472947; doi:10.1007/s00125-015-3585-9)
Supplement: Supplementary file 1 — (PDF 85 kb) [file 125_2015_3585_MOESM1_ESM.pdf]

**ESM Methods:**

**Medical history**

Information on coronary heart disease and stroke at baseline was obtained from self-reported medical diagnosis or from hospital discharge registries; hypertension and hyperlipidemia were based on self-reported diagnosis and/or use of medication. Family history of type 2 diabetes in a first-degree relative was collected in all participants except those from Spain, Italy, Heidelberg (Germany), and Oxford (United Kingdom).

**Statistical analyses:**

Family history of diabetes, waist circumference, and hypertension were not included in the multivariable models because they did not change the risk estimates and were not available for all participants. Dietary factors not included in the models because they did not change the risk estimates were total protein, total fat, vitamin C, vitamin E, glycemic index, and glycemic load.

Effect modification by sex, BMI (<25, 25-<30, ≥30), physical activity (inactive, moderately inactive, moderately active, active), smoking (never, former, current), intake of magnesium, vitamin B1, glycemic index, and glycemic load (≥median versus <median for all dietary variables) was assessed by modeling cross-product terms between these variables and fibre intake and by stratified analyses.

To investigate the robustness of the associations, sensitivity analyses were conducted by excluding participants who reported diseases at baseline (history of stroke (n=261), angina pectoris (n=522), myocardial infarction (n=547), hypertension (n=6,863), or hyperlipidemia (n=4,402). We also excluded the following groups of participants one by one: those with a family history of diabetes (n=3,473 in addition to all participants from Italy, Spain, Oxford (United Kingdom) and Heidelberg (Germany) where family history was not ascertained), those who

developed type 2 diabetes within the first 2 years of follow-up (n=975), and those in the top or bottom 10% of the ratio of energy intake/ energy requirement (n=5,218).

**Meta-analysis**

Publications were extracted independently by two investigators. References from the publications obtained were scanned for additional references. One study [1] was identified from a separate search for another project on *glycemic index, glycemic load* and *diabetes*. When there was more than one paper published from a study we used the most recent publication, unless the previous publication provided more details needed for the dose-response analyses. We excluded the publications from EPIC-Potsdam and EPIC-Netherlands [2, 3] because those data are included in the present study, but results for insoluble and soluble fibre from EPIC-Potsdam [2] are included in this analysis because data on insoluble and soluble fibre intake was not available in the EPIC-InterAct study. We excluded duplicate publications from the Nurses' Health Study [4, 5] and used a publication that provided sufficient information for inclusion in dose-response analyses from that study [6], but the most recent result was used for the high vs. low intake analysis of cereal fibre [5]. We used risk estimates with the most comprehensive adjustment for confounding factors, but for one study which adjusted for inflammatory markers as an exploratory analysis we used the less extensively adjusted model [7]. For some studies that only provided results with fibre as a continuous variable [8, 9] we used data that were obtained previously by contact with the authors for the high vs. low intake analysis [2]. For one study which used the highest intake category as the reference category [10] we used the method by Hamling to convert risk estimates so the lowest category became the reference [11]. For studies which reported results stratified by gender or ethnicity, but not overall, we pooled the results using a fixed effects model before combining with the remaining studies. When the median consumption value per category was not provided, the midpoint of the upper and lower boundaries in each category was assigned. If exposure categories were open-ended or had extreme upper and lower bounds, the width of the

adjacent category was used to derive the upper or lower boundary. Sources of heterogeneity were explored in subgroup analyses and meta-regression analyses stratified by study characteristics. Sensitivity analyses were carried out by excluding one study at a time.

Reference List


1. Sakurai M, Nakamura K, Miura K, et al (2012) Dietary glycemic index and risk of type 2 diabetes mellitus in middle-aged Japanese men. Metabolism 61: 47-55

2. Schulze MB, Schulz M, Heidemann C, Schienkiewitz A, Hoffmann K, Boeing H (2007) Fiber and magnesium intake and incidence of type 2 diabetes: a prospective study and meta-analysis. Arch.Intern.Med. 167: 956-965

3. Sluijs I, van der Schouw YT, van der A DL, et al (2010) Carbohydrate quantity and quality and risk of type 2 diabetes in the European Prospective Investigation into Cancer and Nutrition-Netherlands (EPIC-NL) study. Am J Clin Nutr 92: 905-911

4. Colditz GA, Manson JE, Stampfer MJ, Rosner B, Willett WC, Speizer FE (1992) Diet and risk of clinical diabetes in women. Am.J.Clin.Nutr. 55: 1018-1023

5. Hu FB, Manson JE, Stampfer MJ, et al (2001) Diet, lifestyle, and the risk of type 2 diabetes mellitus in women. N.Engl.J.Med. 345: 790-797

6. Salmeron J, Manson JE, Stampfer MJ, Colditz GA, Wing AL, Willett WC (1997) Dietary fiber, glycemic load, and risk of non-insulin-dependent diabetes mellitus in women. JAMA 277: 472-477

7. Wannamethee SG, Whincup PH, Thomas MC, Sattar N (2009) Associations between dietary fiber and inflammation, hepatic function, and risk of type 2 diabetes in older men: potential mechanisms for the benefits of fiber on diabetes risk. Diabetes Care 32: 1823-1825

8. Hodge AM, English DR, O'Dea K, Giles GG (2004) Glycemic index and dietary fiber and the risk of type 2 diabetes. Diabetes Care 27: 2701-2706

9. Stevens J, Ahn K, Juhaeri, Houston D, Steffan L, Couper D (2002) Dietary fiber intake and glycemic index and incidence of diabetes in African-American and white adults: the ARIC study. Diabetes Care 25: 1715-1721

10. Weng LC, Lee NJ, Yeh WT, Ho LT, Pan WH (2012) Lower intake of magnesium and dietary fiber increases the incidence of type 2 diabetes in Taiwanese. J Formos.Med.Assoc. 111: 651-659

11. Hamling J, Lee P, Weitkunat R, Ambuhl M (2008) Facilitating meta-analyses by deriving relative effect and precision estimates for alternative comparisons from a set of estimates presented by exposure level or disease category. Stat.Med. 27: 954-970

12. Lindstrom J, Peltonen M, Eriksson JG, et al (2006) High-fibre, low-fat diet predicts long-term weight loss and decreased type 2 diabetes risk: the Finnish Diabetes Prevention Study. Diabetologia 49: 912-920
