# Supplementary material for: Dietary fibre and incidence of type 2 diabetes in eight European countries: the EPIC-InterAct Study and a meta-analysis of prospective studies
Source: Diabetologia. 2015 May 29;58(7):1394–408. doi: 10.1007/s00125-015-3585-9 (PMC4472947; doi:10.1007/s00125-015-3585-9)
Supplement: Supplementary file 2 — (PDF 53.9 kb) [file 125_2015_3585_MOESM2_ESM.pdf]

ESM Results

The EPIC-InterAct Study

Sensitivity analyses

In sensitivity analyses with adjustment for all confounders and BMI (model 4), exclusion of participants with cardiovascular disease at baseline (stroke, angina pectoris and heart disease) did not substantially alter the results for total fiber, $HR_{Q4\ vs.\ Q1}$=0.98 (95% CI: 0.85-1.12), nor did exclusion of those who developed type 2 diabetes within the first 2 years of follow-up, HR=0.99 (95% CI: 0.89-1.11), or exclusion of those in the top or bottom 10% of the ratio of energy intake/ energy requirement, HR=1.03 (95% CI: 0.91-1.16). Exclusion of participants with family history of diabetes somewhat strengthened the association, HR=0.82 (95% CI: 0.69-0.97), but this might partly be due to the exclusion of four centers where this information was not available, which by itself gave a HR of 0.88 (95% CI: 0.76-1.02).

Meta-analysis

Subgroup and sensitivity analyses

We conducted several subgroup analyses of total fibre and fibre from different sources. In general these results were consistent across strata, but the number of studies was low in some of these subgroup analyses (**ESM Tables 3 and 4**). There was no evidence of heterogeneity between subgroups in any of the analyses ($p_{heterogeneity} \geq 0.11$) (**ESM Tables 3 and 4**). We were not able to identify an explanation for the high heterogeneity within subgroups in the cereal fibre analyses (**ESM Table 3**). In sensitivity analyses excluding one study at a time the results were not significantly altered for any of the analyses (results not shown). Excluding results from a prospective study within an intervention study (1) in the analysis of total fibre did not alter the results, summary RR=0.92 (95% CI: 0.88-0.96, I^2=15.1%, $p_{heterogeneity=}$0.29) per 10 g/d.

1. Lindstrom J, PeltonenM, Eriksson JG et al (2006) High-fibre, low-fat diet predicts long-term weight loss and decreased type 2 diabetes risk: the Finnish Diabetes Prevention Study. Diabetologia 49: 912–920
