# Supplementary figures and images for: Dietary fibre and incidence of type 2 diabetes in eight European countries: the EPIC-InterAct Study and a meta-analysis of prospective studies
Source: Diabetologia. 2015 May 29;58(7):1394–408. doi: 10.1007/s00125-015-3585-9 (PMC4472947; doi:10.1007/s00125-015-3585-9)

**ESM Figure 1:** Flow-chart of study selection for the meta-analysis

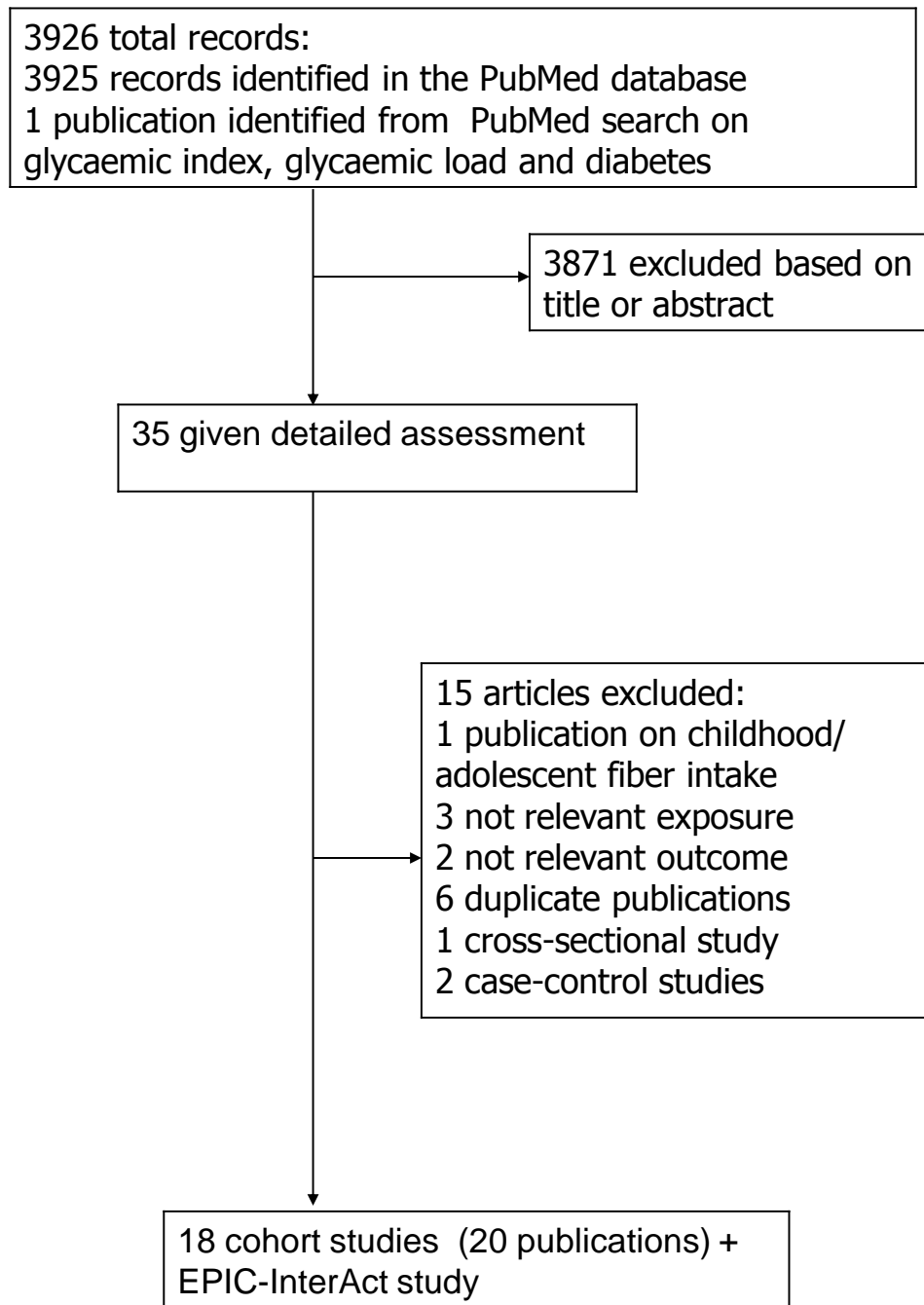

Supplement: Supplementary file 3 — (PDF 51 kb) [file 125_2015_3585_MOESM3_ESM.pdf]
