# Supplementary material for: Dietary fibre and incidence of type 2 diabetes in eight European countries: the EPIC-InterAct Study and a meta-analysis of prospective studies
Source: Diabetologia. 2015 May 29;58(7):1394–408. doi: 10.1007/s00125-015-3585-9 (PMC4472947; doi:10.1007/s00125-015-3585-9)

ESM Figure 2: Dietary fibre (a), cereal fibre (b), fruit fibre (c), vegetable fibre (d) and type 2 diabetes. RRs for the highest vs. the lowest intake. The RR of each study is represented by a square and the size of the square represents the weight of each study to the overall estimate. 95% CIs are represented by the horizontal lines and the diamond represents the overall estimate and its 95% CI

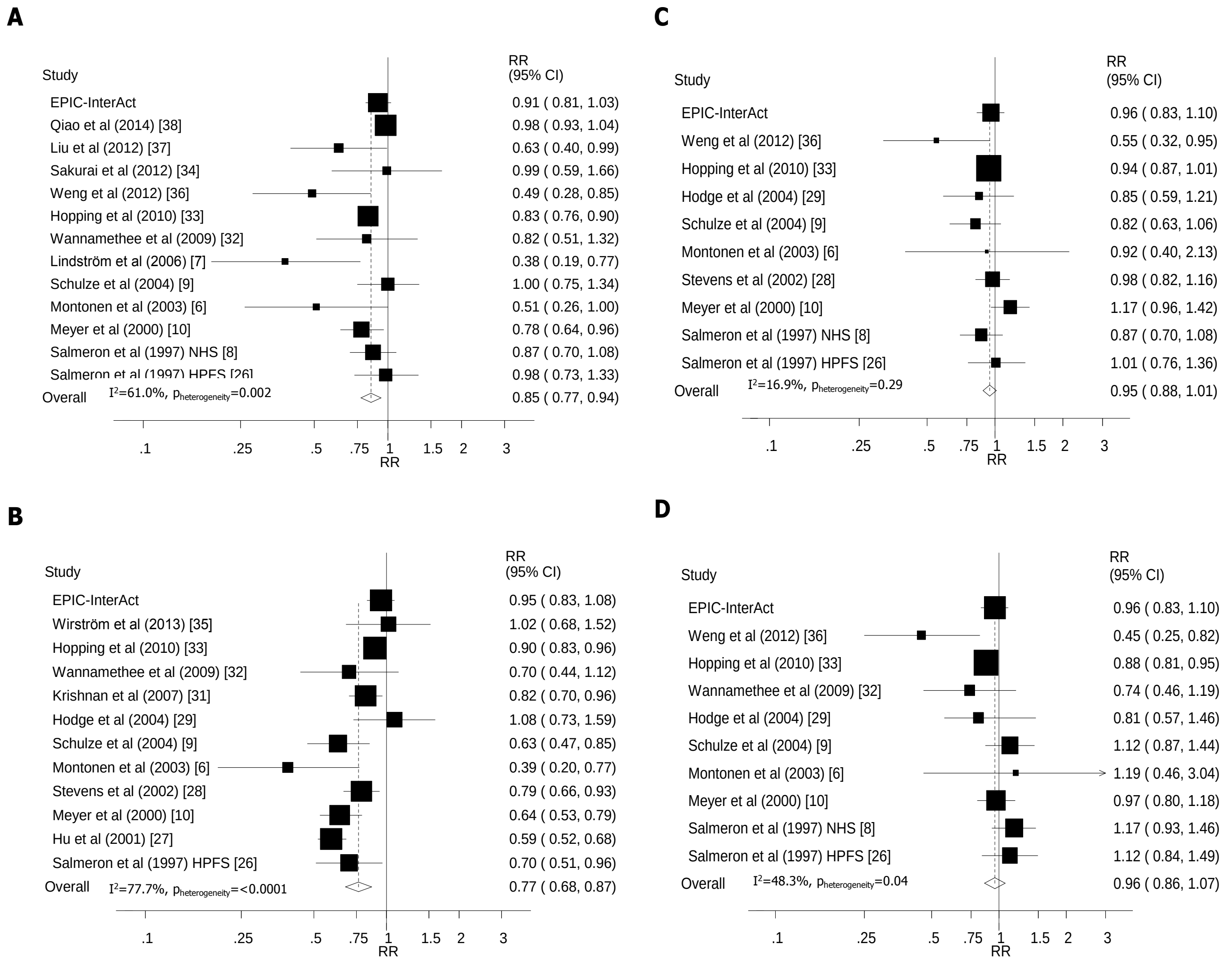

Supplement: Supplementary file 4 — (PDF 145 kb) [file 125_2015_3585_MOESM4_ESM.pdf]
