# Supplementary material for: Dietary fibre and incidence of type 2 diabetes in eight European countries: the EPIC-InterAct Study and a meta-analysis of prospective studies
Source: Diabetologia. 2015 May 29;58(7):1394–408. doi: 10.1007/s00125-015-3585-9 (PMC4472947; doi:10.1007/s00125-015-3585-9)

ESM Figure 3: Soluble (a, b) and insoluble fibre (c, d) and type 2 diabetes, high vs. low (a, c) and linear dose-response (b, d) meta-analysis per 10 g/d. The RR of each study is represented by a square and the size of the square represents the weight of each study to the overall estimate. 95% CIs are represented by the horizontal lines and the diamond represents the overall estimate and its 95% CI

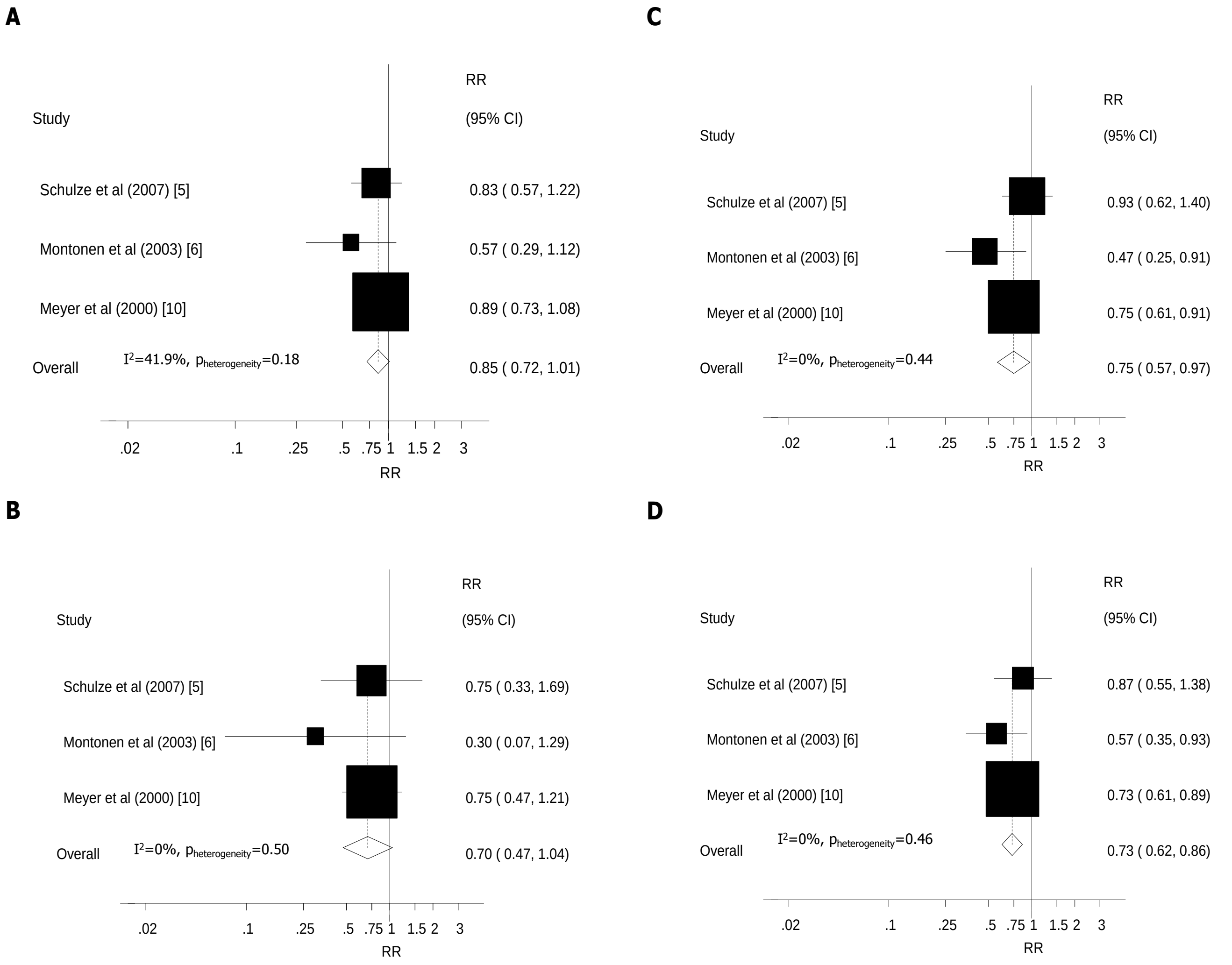

Supplement: Supplementary file 5 — (PDF 170 kb) [file 125_2015_3585_MOESM5_ESM.pdf]
