# Supplementary material for: Dietary fibre and incidence of type 2 diabetes in eight European countries: the EPIC-InterAct Study and a meta-analysis of prospective studies
Source: Diabetologia. 2015 May 29;58(7):1394–408. doi: 10.1007/s00125-015-3585-9 (PMC4472947; doi:10.1007/s00125-015-3585-9)
Supplement: Supplementary file 6 — (PDF 8 kb) [file 125_2015_3585_MOESM6_ESM.pdf]

ESM Figure 4: Funnel plot for meta-analysis of cereal fibre and type 2 diabetes

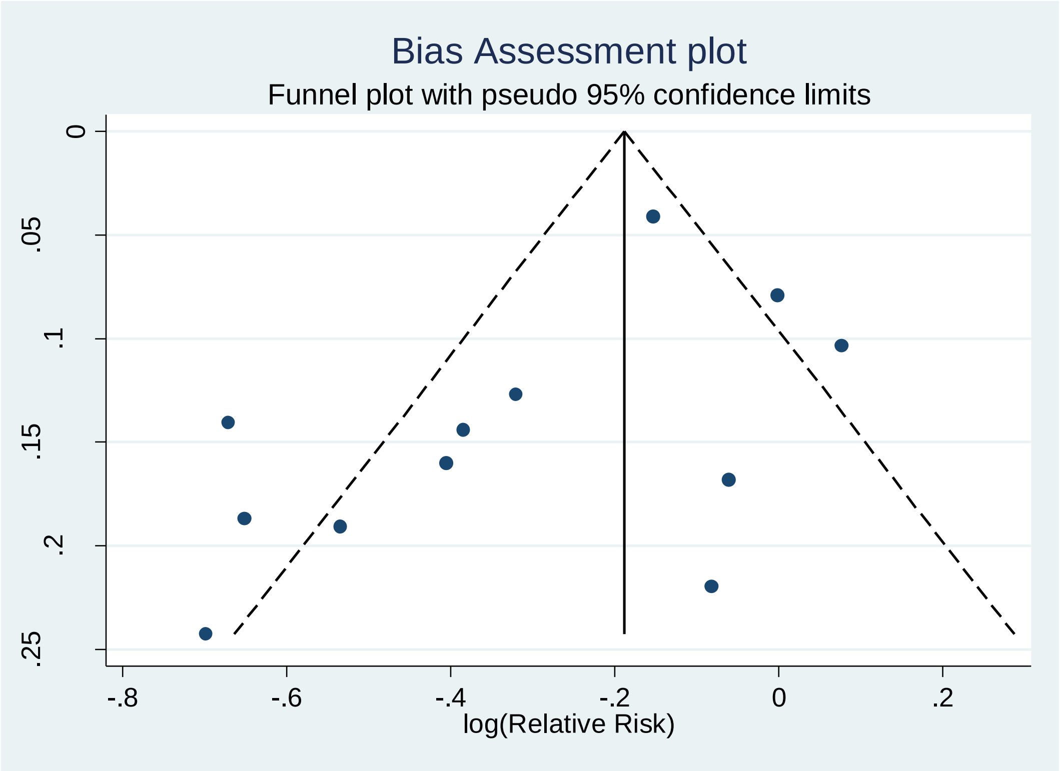

The funnel plot is based on the fixed effects model as recommended by Sterne and Harbord.

Sterne JAC, Harbord RM. Funnel plots in meta-analysis. Stata Journal 2004; 4(2): 127-141.
