# Supplementary material for: Dietary fibre and incidence of type 2 diabetes in eight European countries: the EPIC-InterAct Study and a meta-analysis of prospective studies
Source: Diabetologia. 2015 May 29;58(7):1394–408. doi: 10.1007/s00125-015-3585-9 (PMC4472947; doi:10.1007/s00125-015-3585-9)
Supplement: Supplementary file 7 — (PDF 10 kb) [file 125_2015_3585_MOESM7_ESM.pdf]

ESM Table 1: Mean BMI per quartile of total fibre intake by country

Country	Q1	Q2	Q3	Q4
All	25.8	25.99	26.18	26.16
France	23.44	23.02	23.03	22.53
Italy	25.58	25.45	25.92	26.60
Spain	28.00	28.01	28.34	28.18
UK	25.67	25.29	25.50	24.97
Netherlands	25.24	25.24	25.26	26.23
Germany	25.80	26.00	25.82	25.14
Sweden	25.07	25.37	25.00	24.76
Denmark	26.36	26.30	26.00	25.64
