# Supplementary material for: Dietary fibre and incidence of type 2 diabetes in eight European countries: the EPIC-InterAct Study and a meta-analysis of prospective studies
Source: Diabetologia. 2015 May 29;58(7):1394–408. doi: 10.1007/s00125-015-3585-9 (PMC4472947; doi:10.1007/s00125-015-3585-9)
Supplement: Supplementary file 8 — (PDF 15 kb) [file 125_2015_3585_MOESM8_ESM.pdf]

ESM Table 2. Table of RRs (95% CIs) from nonlinear dose-response analysis

	Fibre		Cereal fibre		Fruit fibre		Vegetable fibre
Quantity	RR (95% CI)	Quantity	RR (95% CI)	Quantity	RR (95% CI)	Quantity	RR (95% CI)
3.4 g/day	1.00	1.74 g/day	1.00	1.0 g/day	1.00	1.25 g/day	1.00
5.0	0.99 (0.95-1.02)	5.0	0.88 (0.84-0.93)	2.0	0.95 (0.89-1.00)	2.0	1.01 (1.00-1.01)
10.0	0.95 (0.86-1.06)	10.0	0.80 (0.74-0.87)	4.0	0.93 (0.87-1.00)	4.0	1.04 (1.01-1.06)
15.0	0.92 (0.79-1.08)	15.0	0.72 (0.65-0.79)	6.0	0.92 (0.86-0.99)	6.0	1.06 (1.02-1.10)
20.0	0.88 (0.73-1.06)	20.0	0.61 (0.51-0.73)	8.0	0.91 (0.85-0.98)	8.0	1.06 (1.00-1.12)
25.0	0.84 (0.68-1.03)	25.0	0.48 (0.34-0.69)	10.0	0.90 (0.83-0.97)	10.0	1.01 (0.95-1.07)
30.0	0.78 (0.64-0.96)	30.0	0.35 (0.19-0.66)	12.0	0.87 (0.80-0.95)	12.0	0.90 (0.84-0.96)
35.0	0.72 (0.59-0.88)			14.0	0.84 (0.74-0.95)	14.0	0.74 (0.66-0.82)
40.0	0.65 (0.53-0.79)						
