# Supplementary material for: Dietary fibre and incidence of type 2 diabetes in eight European countries: the EPIC-InterAct Study and a meta-analysis of prospective studies
Source: Diabetologia. 2015 May 29;58(7):1394–408. doi: 10.1007/s00125-015-3585-9 (PMC4472947; doi:10.1007/s00125-015-3585-9)
Supplement: Supplementary file 9 — (PDF 122 kb) [file 125_2015_3585_MOESM9_ESM.pdf]

ESM Table 3: Subgroup analyses of fibre and sources of fibre and type 2 diabetes risk, dose-response meta-analysis per 10 g/d

		**Total fibre, per 10 g/d**					**Cereal fibre, per 10 g/d**					**Fruit fibre, per 10 g/d**				
		n	RR (95% CI)	I^2 (%)	P_h[a]	P_h[b]	*n*	RR (95% CI)	I^2 (%)	P_h[a]	P_h[b]	*n*	RR (95% CI)	I^2 (%)	P_h[a]	P_h[b]
All studies		15	0.91 (0.87, 0.96)	29.4	0.14		12	0.75 (0.65, 0.86)	75.1	<0.0001		11	0.95 (0.87, 1.03)	31.1	0.15	
Duration of follow-up																
<10 yrs follow-up		11	0.91 (0.84, 0.98)	38.7	0.09	0.85	8	0.68 (0.55, 0.85)	76.9	<0.0001	0.19	7	0.95 (0.82, 1.10)	55.0	0.04	0.65
≥10 yrs follow-up		4	0.92 (0.87, 0.96)	12.8	0.33		4	0.87 (0.76, 1.00)	49.8	0.11		4	0.92 (0.86, 0.99)	0	0.99	
Gender																
Men		4	0.91 (0.87, 0.96)	0	0.88	0.61/ 0.83[c]	3	0.80 (0.64, 1.01)	50.9	0.13	0.46/ 0.33[c]	2	0.95 (0.86, 1.04)	0	0.50	0.79/ 0.79[c]
Women		4	0.92 (0.86, 0.98)	17.4	0.30		6	0.66 (0.52, 0.82)	72.6	0.003		4	0.93 (0.74, 1.16)	63.7	0.04	
Men and women		8	0.89 (0.80, 0.98)	55.9	0.03		5	0.87 (0.72, 1.06)	66.4	0.02		6	0.95 (0.85, 1.05)	17.0	0.30	
Geographic location																
Europe		4	0.82 (0.68, 1.00)	57.8	0.07	0.64	3	0.88 (0.69, 1.12)	61.2	0.08	0.46	2	0.94 (0.77, 1.14)	0	0.85	0.66
America		6	0.92 (0.89, 0.96)	1.8	0.41		7	0.64 (0.52, 0.78)	76.9	<0.0001		6	0.98 (0.87, 1.11)	50.5	0.07	
Asia		2	0.73 (0.58, 0.92)	0	0.63		0					1	0.70 (0.52, 0.94)			
Australia		3	0.94 (0.80, 1.11)	22.4	0.28		2	1.05 (0.87, 1.26)	0	0.51		2	0.95 (0.81, 1.12)	0	0.67	
Number of cases																
Cases <500		8	0.82 (0.71, 0.95)	47.9	0.06	0.30	4	0.90 (0.72, 1.13)	53.3	0.09	0.47	4	0.87 (0.74, 1.03)	13.1	0.33	0.19
Cases 500-<1000		3	0.95 (0.86, 1.05)	0	0.92		3	0.54 (0.43, 0.68)	0	0.85		3	0.87 (0.67, 1.12)	27.2	0.25	
Cases ≥1000		4	0.92 (0.88, 0.98)	39.6	0.17		5	0.76 (0.63, 0.92)	81.0	<0.0001		4	1.00 (0.89, 1.12)	54.7	0.09	
Adjustment for confounding factors																
Alcohol	Yes	8	0.92 (0.86, 0.98)	32.1	0.17	0.74	6	0.68 (0.50, 0.93)	86.8	<0.0001	0.48	7	0.93 (0.80, 1.08)	50.6	0.06	0.78
	No	7	0.90 (0.82, 0.98)	36.8	0.15		6	0.81 (0.73, 0.89)	20.9	0.28		4	0.95 (0.88, 1.01)	0	0.50	

Smoking	Yes	11	0.91 (0.86, 0.97)	33.5	0.13	0.73	11	0.73 (0.61, 0.87)	76.6	<0.0001	0.55	10	0.95 (0.85, 1.06)	34.6	0.13	0.82
	No	4	0.79 (0.57, 1.10)	35.5	0.20		1	0.86 (0.79, 0.93)				1	0.92 (0.85, 1.00)			
Body mass index	Yes	13	0.92 (0.87, 0.97)	35.4	0.10	0.38	11	0.74 (0.64, 0.86)	77.2	<0.0001	0.51	10	0.95 (0.87, 1.04)	37.5	0.11	0.76
	No	2	0.83 (0.69, 1.00)	0	0.77		1	0.92 (0.61, 1.44)				1	0.88 (0.60, 1.30)			
Physical activity	Yes	13	0.92 (0.88, 0.96)	31.6	0.13	0.22	11	0.76 (0.65, 0.88)	76.3	<0.0001	0.71	10	0.95 (0.87, 1.03)	37.8	0.11	0.87
	No	2	0.76 (0.58, 0.98)	0	0.89		1	0.67 (0.49, 0.91)				1	0.75 (0.08, 7.20			
Coffee, caffeine	Yes	1	0.97 (0.78, 1.21)			0.66	1	0.50 (0.31, 0.80)			0.24	1	0.65 (0.40, 1.03)			0.16
	No	14	0.91 (0.86, 0.96)	33.8	0.11		11	0.77 (0.67, 0.88)	74.8	<0.0001		10	0.96 (0.89, 1.04)	24.6	0.22	
Fat	Yes	1	0.95 (0.86, 1.05)			0.57	2	0.86 (0.63, 1.18)	78.1	0.03	0.39	1	0.94 (0.78, 1.14)			0.96
	No	14	0.90 (0.86, 0.96)	33.0	0.11		10	0.72 (0.61, 0.85)	75.7	<0.0001		10	0.95 (0.86, 1.04)	38.0	0.11	
Glycemic load or glycemic index	Yes	2	0.97 (0.78, 1.21)	0	0.91	0.65	2	0.64 (0.45, 0.91)	47.3	0.17	0.40	1	0.65 (0.40, 1.03)			0.16
	No	13	0.91 (0.86, 0.96)	38.8	0.08		10	0.77 (0.66, 0.90)	76.6	<0.0001		10	0.96 (0.89, 1.04)	24.6	0.22	
Magnesium	Yes	2	0.95 (0.87, 1.04)	0	0.87	0.44	2	0.73 (0.37, 1.44)	86.6	0.006	0.80	2	0.83 (0.59, 1.17)	52.5	0.15	0.45
	No	13	0.90 (0.85, 0.95)	37.4	0.09		10	0.74 (0.63, 0.86)	73.3	<0.0001		9	0.96 (0.88, 1.05)	32.9	0.16	
Energy intake	Yes	12	0.92 (0.88, 0.96)	22.9	0.22	0.13	10	0.72 (0.62, 0.84)	79.2	<0.0001	0.28	10	0.95 (0.87, 1.04)	37.5	0.11	0.76
	No	3	0.73 (0.56, 0.97)	13.2	0.32		2	0.93 (0.72, 1.21)	0	0.94		1	0.88 (0.60, 1.30)			

n denotes the number of studies.

a) P for heterogeneity within each subgroup,

b) P for heterogeneity between subgroups with meta-regression analysis,

c) P for heterogeneity between men and women (studies with genders mixed were excluded)
