# Supplementary material for: Dietary fibre and incidence of type 2 diabetes in eight European countries: the EPIC-InterAct Study and a meta-analysis of prospective studies
Source: Diabetologia. 2015 May 29;58(7):1394–408. doi: 10.1007/s00125-015-3585-9 (PMC4472947; doi:10.1007/s00125-015-3585-9)
Supplement: Supplementary file 10 — (PDF 102 kb) [file 125_2015_3585_MOESM10_ESM.pdf]

ESM Table 4: Subgroup analyses of vegetable fibre and type 2 diabetes risk, dose-response meta-analysis per 10 g/d

		**Vegetable fibre, per 10 g/d**				
		n	RR (95% CI)	I^2 (%)	P_h[a]	P_h[b]
All studies		10	0.93 (0.82, 1.05)	43.5	0.07	
Duration of follow-up						
<10 yrs follow-up		6	0.98 (0.82, 1.05)	52.9	0.06	0.21
≥10 yrs follow-up		4	0.88 (0.82, 0.94)	0	0.50	
Gender						
Men		2	0.90 (0.69, 1.16)	58.7	0.12	0.46/
Women		4	0.99 (0.88, 1.11)	17.2	0.31	0.28[c]
Men and women		5	0.78 (0.59, 1.03)	47.0	0.11	
Geographic location						
Europe		2	0.91 (0.69, 1.21)	0	0.81	0.32
America		5	0.99 (0.86, 1.15)	44.6	0.13	
Asia		1	0.52 (0.33, 0.82)			
Australia		2	0.80 (0.47, 1.36)	65.5	0.09	
Number of cases						
Cases <500		4	0.71 (0.47-1.07)	55.7	0.08	0.80
Cases 500-<1000		3	1.14 (0.95-1.38)	0	0.68	
Cases ≥1000		3	0.89 (0.83-0.95)	0	0.81	
Adjustment for confounding factors						
Alcohol	Yes	7	0.97 (0.83, 1.15)	45.3	0.09	0.33
	No	3	0.84 (0.69, 1.03)	12.2	0.32	

Smoking	Yes	9	0.94 (0.80, 1.11)	44.5	0.07	0.75
	No	1	0.88 (0.82, 0.95)			
Body mass index	Yes	9	0.95 (0.84, 1.07)	40.2	0.10	0.18
	No	1	0.58 (0.33, 1.00)			
Physical activity	Yes	9	0.93 (0.82, 1.06)	49.6	0.04	0.87
	No	1	1.16 (0.16, 8.22			
Coffee, caffeine	Yes	1	1.29 (0.93, 1.79)			0.11
	No	9	0.90 (0.80, 1.01)	29.0	0.19	
Fat	Yes	1	0.91 (0.68, 1.21)			0.93
	No	9	0.93 (0.81, 1.08)	49.8	0.04	
Glycemic load or glycemic index	Yes	1	1.29 (0.93, 1.79)			0.11
	No	9	0.90 (0.80, 1.01)	29.0	0.19	
Magnesium	Yes	2	1.07 (0.76, 1.51)	59.6	0.12	0.35
	No	8	0.90 (0.78, 1.03)	37.7	0.13	
Energy intake	Yes	9	0.95 (0.84, 1.07)	40.2	0.10	0.18
	No	1	0.58 (0.33, 1.00)			

n denotes the number of studies.

a) P for heterogeneity within each subgroup,

b) P for heterogeneity between subgroups with meta-regression analysis,

c) P for heterogeneity between men and women (studies with genders mixed were excluded)
